# Adjacent cartilage tissue structure after successful transplantation: a quantitative MRI study using T_2_ mapping and texture analysis

**DOI:** 10.1007/s00330-022-08897-y

**Published:** 2022-06-23

**Authors:** Veronika Janacova, Pavol Szomolanyi, Alexandra Kirner, Siegfried Trattnig, Vladimir Juras

**Affiliations:** 1 High-Field MR Centre, Department of Biomedical Imaging and Image-guided Therapy, Medical University of Vienna, BT32, Lazarettgasse 14, 1090 Vienna, Austria; 2 Institute of Measurement Science, Slovak Academy of Sciences, Bratislava, Slovakia; 3 TETEC Tissue Engineering Technologies AG, Aspenhaustraße 18, 72770 Reutlingen, Germany; 4 CD Laboratory for Clinical Molecular MR Imaging, Vienna, Austria; 5 Austrian Cluster for Tissue Regeneration, Vienna, Austria; 6 Institute for Clinical Molecular MRI in the Musculoskeletal System, Karl Landsteiner Society, Vienna, Austria

**Keywords:** Cartilage, Repair, Texture analysis, Gray-level co-occurrence matrix, Adjacent tissue

## Abstract

**Objectives:**

The aim of this study was to assess the texture of repair tissue and tissue adjacent to the repair site after matrix-associated chondrocyte transplantation (MACT) of the knee using gray-level co-occurrence matrix (GLCM) texture analysis of T_2_ quantitative maps.

**Methods:**

Twenty patients derived from the MRI sub-study of multicenter, single-arm phase III study underwent examination on a 3 T MR scanner, including a T_2_ mapping sequence 12 and 24 months after MACT. Changes between the time points in mean T_2_ values and 20 GLCM features were assessed for repair tissue, adjacent tissue, and reference cartilage. Differences in T_2_ values and selected GLCM features between the three cartilage sites at two time points were analyzed using linear mixed-effect models.

**Results:**

A significant decrease in T_2_ values after MACT, between time points, was observed only in repair cartilage (*p* < 0.001). Models showed significant differences in GLCM features between repair tissue and reference cartilage, namely, autocorrelation (*p* < 0.001), correlation (*p* = 0.015), homogeneity (*p* = 0.002), contrast (*p* < 0.001), and difference entropy (*p* = 0.047). The effect of time was significant in a majority of models with regard to GLCM features (except autocorrelation) (*p* ≤ 0.001). Values in repair and adjacent tissue became similar to reference tissue over time.

**Conclusions:**

GLCM is a useful add-on to T_2_ mapping in the evaluation of knee cartilage after MACT by increasing the sensitivity to changes in cartilage structure. The results suggest that cartilage tissue adjacent to the repair site heals along with the cartilage implant.

**Key Points:**

• *GLCM is a useful add-on to T*_*2*_ *mapping in the evaluation of knee cartilage after MACT by increasing the sensitivity to changes in cartilage structure.*

*• Repair and adjacent tissue became similar to reference tissue over time.*

*• The results suggest that cartilage tissue adjacent to the repair site heals along with the cartilage implant.*

**Supplementary Information:**

The online version contains supplementary material available at 10.1007/s00330-022-08897-y.

## Introduction

The articular surfaces of the knee joint are covered by hyaline cartilage, which can withstand high repeated pressure and provides low-friction surfaces for joint motion [1, 2]. The prevalence of cartilage defects in the knee is estimated to be approximately 12% of the overall population [3]. In young adults, these defects are mostly the result of sport injuries, other trauma, or mechanical malalignment of the joint [4]. Subsequent damage to the chondrocytes leads to degeneration and death of cells, cartilage matrix degradation, decreased production of proteoglycans, increased water content, and loss of zonal architecture [2]. In cases where conservative treatment is not effective, bone marrow stimulating techniques (MFx) and cartilage restoration techniques, such as matrix-associated autologous chondrocyte transplantation (MACT), are often used [5–8]. Previous studies have shown that the outcome of cartilage repair surgery can vary from hyaline-like tissue to mixed fibrous-hyaline tissue or fibrous tissue with inferior biomechanical properties often seen after MFx [9–11]. Furthermore, the tissue adjacent to the cartilage repair site might be affected by degenerative changes in the perilesional zone caused by insufficient defect preparation [12] or suturing of articular cartilage [13].

Early stages of these changes in adjacent cartilage tissue are often undetectable on routine morphological scans. Quantitative MRI, especially T_2_ mapping, which is sensitive to collagen matrix organization and water content [14, 15], has been proven to be a reliable non-invasive diagnostic technique [16, 17], as degradation of cartilage matrix leads to increased water mobility and subsequent T_2_ elevation [15]. Differences in T_2_ distributions are often visible on T_2_ maps, which suggests that the analysis of spatial T_2_ distribution might be a valuable tool for assessing changes of repair site and the surrounding tissue during standard post procedure follow-ups. Gray-level co-occurrence matrix (GLCM) texture analysis [18] has been explored in recent years in the context of detecting early osteoarthritic (OA) changes [19–24]. The GLCM determines the co-occurrence of different signal intensities in a specific offset in the image, creating a co-occurrence matrix from which different quantitative features can be extracted. GLCM features (second-order image statistics) provide information about linear and quadratic relationships between pixel pairs. There are various properties of the GLCM calculation. The displacement distance (d) determines the distance between neighboring pixels [18, 25], the angle (direction) at which GLCM is calculated, and the number of gray levels. However, in most cases, the quantization into 16 gray levels is sufficient [25]. The angle (direction) at which GLCM is calculated is an important property to consider, because the structure of cartilage is not homogenous in all directions. Since the region of interest (ROI) might not be rectangular, ROI flattening was implemented in various studies to improve the reproducibility of GLCM calculation [21, 26]. The aim of this study was to assess the properties of the repair site after MACT and its adjacent cartilage tissue and compare these to healthy cartilage using quantitative MRI techniques (T_2_ mapping) and advanced image texture analysis techniques, such as GLCM.

## Method

### Study design and patient baseline characteristics

The institutional review board or independent ethics committee at each site approved the study, and each participant gave written, informed consent.

Our cohort consisted of a subgroup of patients who participated in the MRI sub-study of a prospective, multicenter, single-arm phase III study evaluating efficacy and safety of MACT using NOVOCART® Inject (TETEC Tissue Engineering Technologies AG). Males and females 18 to 65 years of age (or ≥ 14-year-old minors with a closed epiphyseal growth plate) with focal cartilage defects of the femoral condyle, trochlea, patella, or tibial plateau (ICRS grade III or IV) were eligible for enrollment. Two defects and prior failed cartilage repair of the index lesion were allowed.

Three study sites participated in MRI examination of patients, which was performed 12 and 24 months after MACT.

Patient demographic and baseline characteristics of the 25 patients (with overall 30 cartilage defects) who participated in the MRI sub-study are summarized in Table 1. In terms of lesion etiology, trauma and focal degeneration were reported for 46.7% of the defects each, and 6.7% of the defects were caused by osteochondritis dissecans (OCD). Five patients from the MRI cohort were excluded, due to a small defect size, resulting in 20 evaluable patients (with overall 23 lesions). Detailed information about postoperative recovery and rehabilitation of all patients can be found in Niemeyer et al. [27].

**Table 1:** Patient demographic and defect characteristics

	All patients (*N* = 25)
Sex, *n* (%)	
Male	17 (68.0)
Female	8 (32.0)
Age (years), mean ± SD	39.6 ± 13.5 (15–60)
Number of defects per patient, *n* (%)	
One defect	20 (80)
Two defects	5 (20)
Defect location, *n* defects (%)	
Femoral condyle	22 (73.3)
Patellofemoral	7 (23.3)
Tibial plateau	1 (3.3)
ICRS grade, *n* defects (%)	
3	24 (80.0)
4	4 (13.3)
Lesion etiology, *n* defects (%)	
Traumatic	14 (46.7)
OCD	2 (6.7)
Focal degenerative	14 (46.7)
Defect size (cm^2^), mean ± SD	
All lesions	5.5 ± 1.9 (1.0–9.0)
Larger lesion^1,2^	6.0 ± 1.6 (4.0–9.0)
Total^3^	6.6 ± 2.3 (4.0–12.5)

*n*, number of patients; n defects, number of defects; *SD*, standard deviation; *ICRS*, International Cartilage Repair Society; *OCD*, osteochondritis dissecans

^1^Lesions were classified into larger and smaller lesions, i.e., in patients with two lesions, the classification was based on the size of the respective lesions, while in patients with one lesion only, this lesion was classified as the larger lesion

^2^Total number of larger lesions was 25

^3^All lesions per patient added to one single value

### MRI examination

MRI examinations consisted of morphological imaging protocols (three-dimensional proton density-weighted GRE sequence, and two-dimensional proton-density, T_1_- and T_2_-weighted fast spin echo sequences) and T_2_ mapping multi-echo spin-echo sequence. T_2_ maps were acquired using the parameters listed in Table 2 for each MRI site. T_2_ mapping was performed centrally in MATLAB 9.6 (MathWorks) using mono-exponential, two-parametric decay fitting.

**Table 2:** T_2_ mapping sequence parameters for each of the three sites participating in this study

Site		1	2	3
Scanner		Achieva (Philips) 3 T	Achieva (Philips) 3 T	MAGNETOM Skyra (Siemens) 3 T
Coil		8-channel knee	16-channel knee	15-channel knee
Sequence		Multi-echo spin-echo	Multi-echo spin-echo	Multi-echo spin-echo
Orientation plane		Sagittal	Sagittal	Sagittal
Slice thickness (mm)		3	3	3
Slice spacing (mm)		3.3	3.6	3.3
Repetition time (ms)		2000	2000	2000
Echo times (ms)				
Number	1	12.5	12.5	12.5
	2	25	25	25
	3	37.5	37.5	37.5
	4	50	50	50
	5	62.5	62.5	62.5
	6	75	75	75
	7	87.5	87.5	87.5
	8	100	100	100
Averages		1	1	1
Acquisition matrix		268 × 320	268 × 320	320 × 256
Field-of-view (cm)		16 × 16	16 × 16	16 × 16
Total acquisition time		7 min 52 s	9 min 52 s	8 min 4 s

### Image evaluation

Morphological images were assessed by an experienced radiologist using the MOCART 1.0 score (Magnetic Resonance Observation of Cartilage Repair Tissue) for semi-quantitative assessment of the repair tissue [28].

**Fig. 1:**
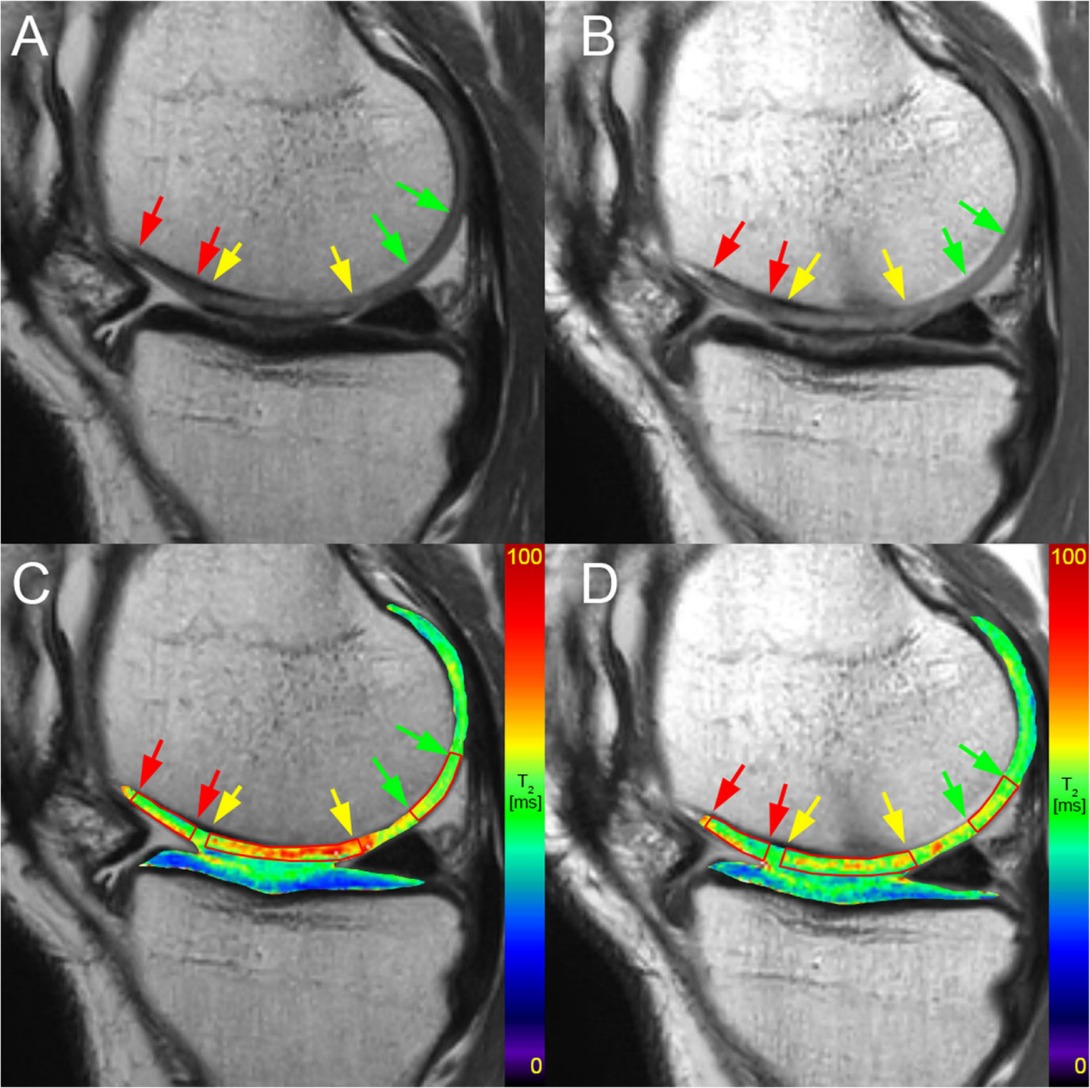
ROI selection on T_2_-weighted MR images at 12 months and 24 months (**A** and **B**, respectively). ROIs were transferred onto the T_2_ maps (red delineation) at 12 and 24 months (**C** and **D**, respectively). Yellow arrows point toward the lesion site boundaries, red arrows point toward the adjacent tissue boundaries and green arrows point toward the reference tissue boundaries

Regions of interest (ROIs) were defined by an experienced radiologist on two or three consecutive slices of the T_2_ mapping sequence based on morphological images using JiveX (Visus). The number of slices depended on the size of the lesion. For each slice, repair cartilage, adjacent cartilage, and a reference cartilage were selected. Adjacent tissue was selected immediately next to the repair tissue. Cartilage located at least 10 mm from the edge of the repair tissue was selected as a reference. ROIs were placed in the same location of the cartilage at both time points. ROIs were subsequently transferred onto corresponding slices of T_2_ maps and processed in MATLAB 9.6 (MathWorks) (Fig. 1). Mean T_2_ was calculated and averaged through the slices, ROI-wise, resulting in three T_2_ values per patient (one for repair cartilage, one for adjacent cartilage, and one for reference cartilage).

### GLCM analysis

Inclusion criterion for GLCM was the number of pixels in an ROI greater than fifteen. Using an in-house-written script in MATLAB, ROIs were rotated and flattened (MATLAB function “imrotate”), quantized into 16 gray levels, and consecutive GLCM analysis was computed with an offset of 0° (direction parallel to the cartilage surface) and a step of length 1 (considering a pixel and its immediate neighbor). Using the *GLCM_features1* function from the MATLAB Repository [29], 20 quantitative features were extracted (*autocorrelation*, *cluster prominence*, *cluster shade*, *contrast*, *correlation*, *difference entropy*, *difference variance*, *dissimilarity*, *energy*, *entropy*, *homogeneity*, *information measure*, *information measure of correlation 2*, *inverse difference moment normalized*, *inverse difference normalized INN*, *maximum probability*, *sum average*, *sum entropy*, *sum of squares*, *sum variance*). GLCM feature values were averaged through the slices, ROI-wise, resulting in three values (for each ROI) for every feature per patient. Texture feature reproducibility and optimization of GLCM setup (offset, number of gray levels, and step) for focal cartilage lesions were performed prior to this study and published elsewhere [30, 31].

### Statistical methods

All statistical analyses were performed using R version 4.0.5 (R Foundation for Statistical Computing) in RStudio version 1.4.1106 (Rstudio, PBC). T_2_ and GLCM feature values were averaged for every ROI type. The Shapiro-Wilk normality test was used to assess the normality of the examined variables. The Wilcoxon signed-rank test was used to determine the significance of differences in mean T_2_ values and GLCM features between 12 and 24 months separately for each ROI. Spearman’s rank correlation coefficients were calculated. Linear mixed models with a random slope and random intercept were fitted using function *lmer* from the R package *lme4 version 1.1-2* [32]*.* In all models, we considered time point and ROI type as fixed effects and we allowed individual intercepts for each patient with the slopes of the variable *ROI type* varying by patient. A fixed-effects structure was determined using likelihood ratio tests between models. The interaction between fixed effects was specified only in the model that explored the variable *mean T*_*2*_. Final models were fitted with restricted maximum likelihood (REML). The variables *correlation* and *contrast* had to be transformed to meet model assumptions. Repair tissue at 12 months was set as the baseline in each model and we report fixed effects that describe the course of T_2_ and GLCM features in time.

*p*–values lower than 0.05 were considered statistically significant.

## Results

The mean MOCART score increased from 74.6 ± 12.2 at 12 months to 88.7 ± 8.8 at 24 months (for details, see Table 3). A significant decrease in mean T_2_ values between the two post-surgical time points (12 and 24 months) was observed only for repair cartilage (*p* < 0.001). In terms of GLCM, significant changes (*p* < 0.05) over time were observed for repair cartilage in the following features: *contrast, correlation*, *difference entropy*, *difference variance*, *dissimilarity*, *homogeneity*, *information measure*, *information measure of correlation 2*, *inverse difference moment normalized*, and *inverse difference normalized*. Similarly, significant changes (*p* < 0.05) from 12 to 24 months were found in the following GLCM features in tissue adjacent to the repair site: *contrast*, *correlation*, *difference entropy*, *difference variance*, *dissimilarity*, *homogeneity*, *information measure*, *inverse difference moment normalized*, and *inverse difference normalized*. The changes in adjacent cartilage were similar to those for repair cartilage. In reference to cartilage, *autocorrelation*, *sum average*, *sum of squares*, and *sum variance* were significantly different between 12 and 24 months (*p* < 0.05). Mean absolute values with standard deviations and mean differences between time points are specified in Table 4. We found a moderate correlation between MOCART and autocorrelation (0.52), cluster shade (−0.53), sum of squares (0.53), sum average (0.53), and sum variance (0.51).

**Table 3:** MOCART values at 12 and 24 months after surgery for each repair site

Repair site #	12 months	24 months
1	70	90
2	80	80
3	65	80
4	75	90
5	85	90
6	75	80
7	100	100
8	85	90
9	65	100
10	65	85
11	90	95
12	85	95
13	60	95
14	70	90
15	90	100
16	65	85
17	85	95
18	65	80
19	85	90
20	55	75
21	55	65
22	80	95
23	65	95
Mean	74.6	88.7
Standard deviation	12.2	8.8

**Table 4:** Differences in T_2_ values and GLCM features measured at 12 months and 24 months after cartilage repair

	Repair tissue				Adjacent tissue				Reference tissue			
Feature	12M	24M	12M–24M	*p*–value	12M	24M	12M–24M	*p*–value	12M	24M	12M–24M	*p*–value
T_2_	56.6 ± 8.7	50.77 ± 7.12	5.82 ± 6.48	**< 0.001**	53.1 ± 8.97	51.98 ± 10.21	1.13 ± 5.75	0.50	54.33 ± 8.5	53.49 ± 7.26	0.84 ± 5.4	0.45
Autocorrelation	74 ± 35.38	72.03 ± 35.84	2.03 ± 25.02	0.52	107.51 ± 35.71	104.56 ± 34.57	2.97 ± 25.8	0.96	128.12 ± 34.32	117.71 ± 34.55	10.42 ± 21.65	**0.02**
Cluster prominence	1082.11 ± 1517.78	1029.42 ± 1093.07	50.64 ± 1093.92	0.78	805.54 ± 946.03	814.64 ± 947.04	−7.97 ± 607.6	0.89	379.31 ± 380.32	431.19 ± 425.25	−51.88 ± 341.01	0.18
Cluster shade	48.45 ± 81.47	48.06 ± 64.84	0.41 ± 56.63	0.71	29.06 ± 57.56	32.92 ± 62.2	−3.76 ± 42.84	1.00	0.18 ± 19.83	6.62 ± 25.11	−6.44 ± 15.77	0.05
Contrast	1.34 ± 0.81	1.81 ± 1.13	−0.48 ± 0.71	**< 0.001**	1.53 ± 0.95	1.99 ± 1.23	−0.45 ± 0.71	**0.01**	2.11 ± 1.51	2.32 ± 1.57	−0.21 ± 0.76	0.17
Correlation	0.81 ± 0.12	0.76 ± 0.16	0.06 ± 0.09	**< 0.001**	0.79 ± 0.16	0.73 ± 0.19	0.05 ± 0.1	**0.02**	0.63 ± 0.29	0.61 ± 0.28	0.02 ± 0.12	0.33
Difference entropy	1.05 ± 0.2	1.15 ± 0.23	−0.1 ± 0.14	**< 0.001**	1.07 ± 0.22	1.17 ± 0.24	−0.1 ± 0.14	**< 0.001**	1.18 ± 0.26	1.23 ± 0.24	−0.05 ± 0.14	0.09
Difference variance	1.34 ± 0.81	1.81 ± 1.13	−0.48 ± 0.71	**< 0.001**	1.53 ± 0.95	1.99 ± 1.23	−0.45 ± 0.71	**0.01**	2.11 ± 1.51	2.32 ± 1.57	−0.21 ± 0.76	0.17
Dissimilarity	0.75 ± 0.28	0.88 ± 0.33	−0.14 ± 0.2	**0.01**	0.86 ± 0.35	0.98 ± 0.37	−0.12 ± 0.22	**0.01**	1.03 ± 0.45	1.08 ± 0.4	−0.05 ± 0.22	0.34
Energy	0.07 ± 0.03	0.07 ± 0.04	0.00 ± 0.03	0.75	0.07 ± 0.03	0.07 ± 0.04	0.01 ± 0.03	0.33	0.07 ± 0.03	0.06 ± 0.03	0.00 ± 0.02	0.18
Entropy	3.08 ± 0.34	3.15 ± 0.4	−0.08 ± 0.32	0.36	2.92 ± 0.3	3.02 ± 0.36	−0.1 ± 0.31	0.10	3.02 ± 0.33	3.09 ± 0.37	−0.08 ± 0.34	0.22
Homogeneity	0.7 ± 0.08	0.67 ± 0.09	0.03 ± 0.05	**0.01**	0.66 ± 0.1	0.63 ± 0.1	0.03 ± 0.06	**0.07**	0.62 ± 0.11	0.61 ± 0.09	0.01 ± 0.06	0.48
Information measure	−0.39 ± 0.07	−0.34 ± 0.08	−0.05 ± 0.07	**< 0.001**	−0.44 ± 0.08	−0.39 ± 0.07	−0.04 ± 0.07	**0.02**	−0.32 ± 0.12	−0.31 ± 0.12	−0.01 ± 0.07	0.64
Information measure of correlation 2	0.86 ± 0.12	0.84 ± 0.11	0.03 ± 0.06	**0.04**	0.88 ± 0.12	0.87 ± 0.11	0.02 ± 0.05	0.15	0.8 ± 0.12	0.8 ± 0.13	0.00 ± 0.07	0.89
Inverse difference moment normalized	0.99 ± 0.00	0.99 ± 0.00	0.00 ± 0.00	**< 0.001**	0.99 ± 0.00	0.99 ± 0.01	0.00 ± 0.00	**0.01**	0.99 ± 0.01	0.99 ± 0.01	0.00 ± 0.00	0.16
Inverse difference normalized INN	0.96 ± 0.02	0.95 ± 0.02	0.01 ± 0.01	**0.01**	0.95 ± 0.02	0.94 ± 0.02	0.01 ± 0.01	**0.02**	0.94 ± 0.02	0.94 ± 0.02	0.00 ± 0.01	0.38
Maximum probability	0.15 ± 0.06	0.15 ± 0.07	0.00 ± 0.06	0.78	0.15 ± 0.06	0.14 ± 0.08	0.01 ± 0.06	0.46	0.14 ± 0.04	0.13 ± 0.06	0.01 ± 0.04	0.10
Sum average	16.23 ± 4.46	15.94 ± 4.72	0.29 ± 3.38	0.58	20.03 ± 4.05	19.78 ± 3.88	0.24 ± 2.95	0.82	22.18 ± 3.49	21.21 ± 3.44	0.97 ± 2.21	**0.04**
Sum entropy	2.51 ± 0.23	2.48 ± 0.24	0.03 ± 0.21	0.50	2.43 ± 0.27	2.42 ± 0.25	0.01 ± 0.2	0.87	2.36 ± 0.29	2.38 ± 0.32	−0.02 ± 0.24	0.21
Sum of squares	74.94 ± 35.29	72.85 ± 36.25	2.1 ± 25.02	0.48	109.75 ± 37.42	106.32 ± 34.9	3.46 ± 25.74	0.92	130.37 ± 35.1	118.66 ± 35.66	11.71 ± 21.82	**0.01**
Sum variance	222.12 ± 119.84	216.06 ± 119.25	6.36 ± 83.31	0.50	340.97 ± 126.88	330.69 ± 122.31	10.29 ± 88.52	0.99	416.07 ± 125.91	378.85 ± 129.29	37.22 ± 81.57	**0.02**

Values in bold indicate statistically significant results.

**Fig. 2:**
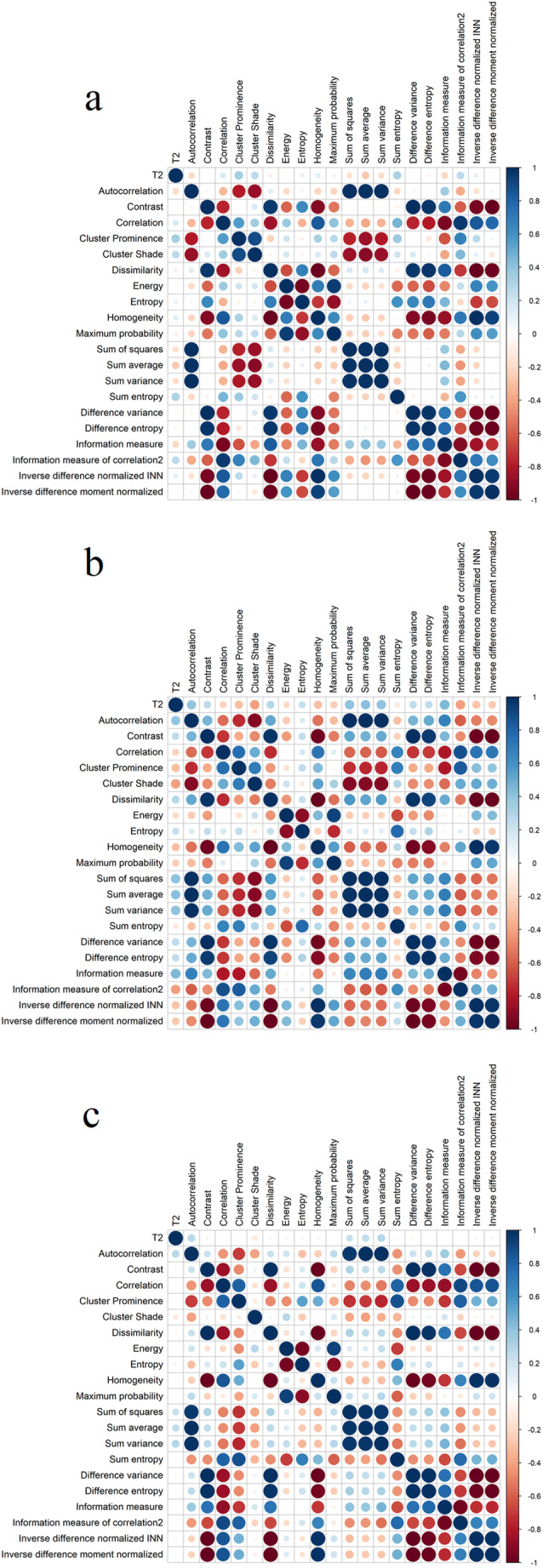
Cross-correlation matrix of GLCM features at 12 months in (**a**) repair tissue, (**b**) adjacent tissue, and (**c**) reference tissue. Some features are highly correlated. For example, contrast and dissimilarity are calculated nearly identically, but contrast uses a weight of *(i-j)*^*2*^ and dissimilarity uses a weight of *(i-j)*, where *i* and *j* are gray levels in rows and columns of the GLCM matrix, respectively [29, 40]. The two measures contain essentially the same information, therefore analysis of only one was sufficient

The following variables were modelled by means of linear-mixed effects models: *mean T*_*2*_, *autocorrelation*, *correlation*, *homogeneity*, *contrast*, and *difference entropy*. Variables were chosen based on results from paired test results and correlations between features (Fig. 2). Because many GLCM features are highly correlated with each other, we analyzed only those that were easily interpretable in the context of cartilage texture and that satisfied model assumptions.

**Fig. 3:**
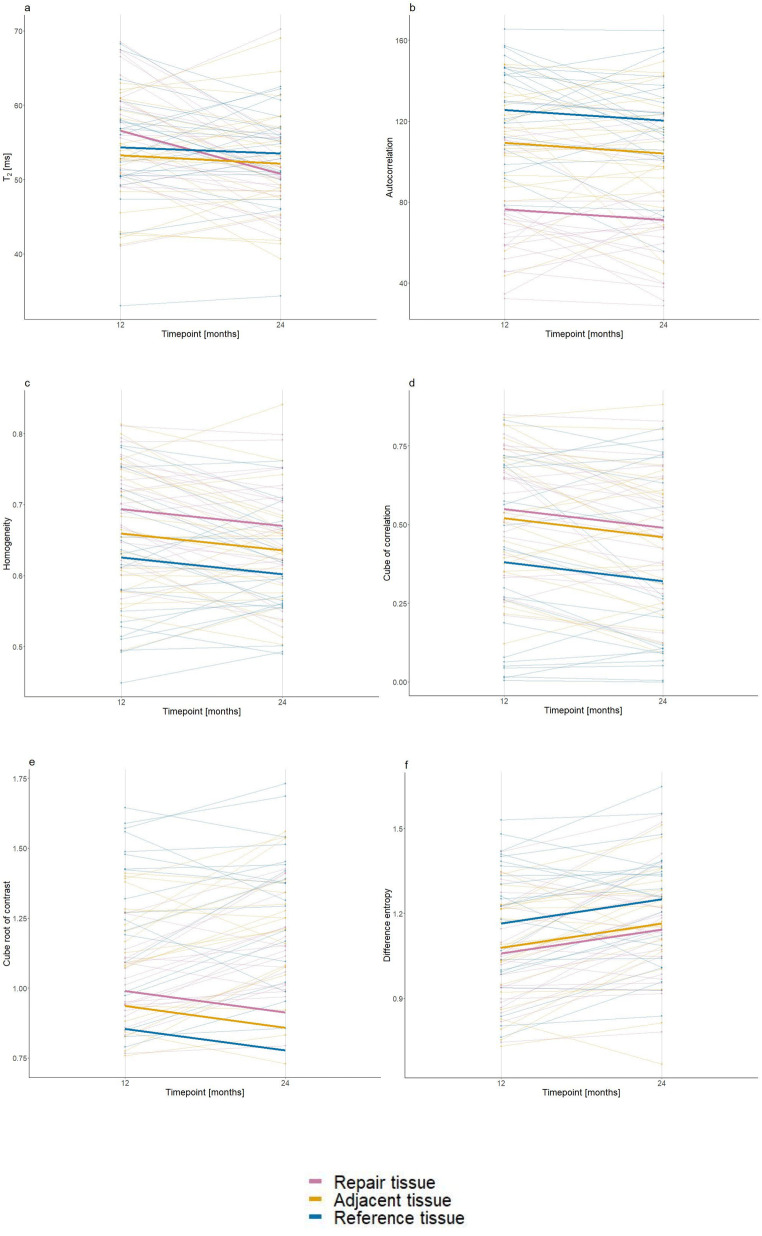
Plot representing a change of mean estimated values T_2_ and GLCM features (bold lines). Paired values for each case, color-coded by ROI, are represented by faded lines in the background. Interaction between time and tissue type in the case of T_2_ is clearly visible (**a**). Similarity between repair and adjacent tissue is visible in the case of the autocorrelation (**b**), cube of correlation (**d**), cube root of contrast (**e**), and difference entropy (**f**) Lines representing mean estimates are evenly spaced in the case of the homogeneity (**c**), representing difference in homogeneity between all three cartilage regions

Our model confirmed a significant (*p* < 0.001) estimated decrease of T_2_ of −5.82 (−8.23 to −3.41) ms from 12 to 24 months after surgery. The rate of decrease was different for each ROI (significant interaction between adjacent tissue and time, and reference tissue and time), showing a more stable mean T_2_ value compared to repair cartilage (Fig. 3a).

The effect of time on autocorrelation was not significant (*p* = 0.08). Adjacent tissue (*p* < 0.001) and reference tissue (*p* < 0.001) had significantly higher mean values compared to repair tissue.

Homogeneity decrease in time was significant (*p* = 0.001). Adjacent tissue (*p* = 0.027) and reference tissue (*p* = 0.002) had significantly lower mean values compared to repair tissue. (Fig. 3c).

Models of the cube of correlation, cube root of contrast, and difference entropy showed a significant effect (*p* < 0.05) of time and a significant difference (*p* < 0.05) between repair and reference tissue. However, the difference between repair and adjacent tissue was not significant.

Summary tables of all models and estimated values can be found in the Supplementary material.

## Discussion

The purpose of this study was to evaluate the T_2_ relaxation time and GLCM texture features in knee cartilage treated with MACT. We were specifically interested in MACT transplant maturation and changes in cartilage tissue adjacent to the repair site (Table 5).

**Table 5:** Fixed effects of linear mixed-effects models describing the change in mean T_2_ and selected GLCM between 12 and 24 months after MACT in different parts of cartilage

Mean T_2_				Autocorrelation				Homogeneity				Correlation^3^				∛Contrast				Difference entropy			
*Predictors*	*Estimates*	*CI*	*p*–value	*Predictors*	*Estimates*	*CI*	*p*–value	*Predictors*	*Estimates*	*CI*	*p*–value	*Predictors*	*Estimates*	*CI*	*p*–value	*Predictors*	*Estimates*	*CI*	*p*–value	*Predictors*	*Estimates*	*CI*	*p*–value
Intercept* (T_2_)	56.59	53.96 to 59.22	**< 0.001**	Intercept* (Autocorrelation)	76.37	65.57 to 87.17	**< 0.001**	Intercept* (Homogeneity)	0.69	0.66 to 0.72	**< 0.001**	Intercept* (Correlation^3^)	0.55	0.47 to 0.63	**< 0.001**	Intercept* (∛Contrast)	0.99	0.93 to 1.05	**< 0.001**	Intercept* (Difference entropy)	1.06	0.99 to 1.13	**< 0.001**
Time**	−3.34	−7.53 to 0.85	0.118	Time**	−5.14	−10.84 to 0.56	0.077	Time**	−0.02	−0.04 to −0.01	**0.001**	Time**	−0.06	−0.09 to −0.03	**< 0.001**	Time**	−0.08	−0.11 to −0.05	**< 0.001**	Time**	0.08	0.05 to 0.12	**< 0.001**
Adjacent tissue	−2.26	−5.54 to 1.03	0.178	Adjacent tissue	32.82	22.34 to 43.30	**< 0.001**	Adjacent tissue	−0.03	−0.06 to −0.004	**0.027**	Adjacent tissue	−0.03	−0.13 to 0.07	0.56	Adjacent tissue	−0.05	−0.11 to 0.01	0.075	Adjacent tissue	0.02	−0.05 to 0.09	0.546
Reference tissue	−5.82	−8.23 to −3.41	**< 0.001**	Reference tissue	49.11	35.40 to 62.83	**< 0.001**	Reference tissue	−0.07	−0.11 to −0.02	**0.002**	Reference tissue	−0.17	−0.30 to −0.03	**0.015**	Reference tissue	−0.14	−0.21 to −0.06	**< 0.001**	Reference tissue	0.11	0.001 to 0.21	**0.047**
Adjacent tissue: Time*	4.69	1.29 to 8.10	**0.007**	–	–	–	–	–	–	–	–	–	–	–	–	–	–	–	–	–	–	–	–
Reference tissue: Time*	4.98	1.58 to 8.39	**0.004**	–	–	–	–	–	–	–	–	–	–	–	–	–	–	–	–	–	–	–	–

*Repair tissue at 12 months

**Time point 24 months

Values in bold indicate statistically significant results.

Elevated T_2_ relaxation time in cartilage is associated with degeneration or trauma and its decrease over time signals changes in cartilage structure [19, 33]. It has been shown that variation of T_2_ in healthy hyaline cartilage was highly correlated with collagen anisotropy fibril angle [34]. Randomly oriented collagen fibers (e.g., in the superficial cartilage zone) allow more mobility of protons, and thus, higher T_2_ relaxation times were found [35, 36]. Early in the maturation process, the MACT graft might have a fluid-like appearance, because the repair tissue is initially poorly organized and highly water-permeable [35–37]. It has been hypothesized that a decrease in T_2_ is connected to the reorganization of the matrix structure and a decrease in free water [35].

In our study, the analysis showed a significant decrease in mean T_2_ between 12 and 24 months only in repair cartilage (∆ = 5.82 ± 6.48, *p* < 0.001). Decreased T_2_ values in combination with a high morphological score (MOCART) indicated a favorable clinical outcome, which is in correspondence with the clinical results of this study published by Niemeyer et al. [27]. A decrease in the mean T_2_ value of a selected ROI is very general and represents only one mean quantitative value for the whole area without providing information about changes in cartilage texture; therefore, texture analysis might be a useful add-on. GLCM features are statistical texture features that describe relationships between individual pixels [18]—information that is lost when comparing mean T_2_ values. These features are highly correlated; hence, the selection of the most fitting features must be considered. This can be done either automatically using an algorithm, or manually, based on correlation and cluster analysis and an understanding of the image intended for analysis [38].

In our study, we used quantization, a process in which a range of values is compressed to a single quantum [39]. If the variance of T_2_ values is large, similar values fall into one quantization bin, which results in larger areas of one gray level. In tissues with a smaller variance of T_2_ values, quantization results in greater disparity in gray levels. This is the reason the texture of reference cartilage is richer. Moreover, the variability of T_2_ values at 12 months among patients is high, which leads to high variability of features. Thus, the specific feature values might not be as important as the direction of differences between ROIs and the two time points. Since our cohort consisted of comparatively young individuals with focal cartilage defects, we can consider our reference to be healthy cartilage tissue.

We also found significant differences in autocorrelation, sum average, sum of squares, and sum variance in reference tissue between 12 and 24 months (Table 2). These features are linked to gray-level dispersion, which is higher in reference tissue. However, the features that are linked to the presence of edges or disorderliness were not significantly different compared to the other two areas. A possible explanation could be a subsidiary improvement of healthy cartilage, with the direction of change in these features the same as that in repair and adjacent tissue, although not significant.

Our analysis of GLCM features showed that autocorrelation [39] does not change between 12 and 24 months, but is significantly higher in adjacent and reference tissue compared to repair tissue, suggesting more prominent patterns and possibly the normal collagen fiber network in healthy reference cartilage. Concerning correlation, adjacent tissue was more similar to repair cartilage, but values approached those of the reference tissue between 12 and 24 months. The linear relationship between pixels is more predictable in repair cartilage and reveals a more uniform texture compared to healthy cartilage.

Images with more uniform gray levels result in a higher overall value of homogeneity [18]. Homogeneity was lower in adjacent (*p* = 0.027) and reference tissue (*p* = 0.002) compared to the repair site. In combination with contrast and difference entropy [18, 40], which both showed significant differences only between repair and reference cartilage, we see adjacent tissue might have been more similar to repair tissue than to reference cartilage, or is between those two, texture-wise.

After transforming contrast to −1/3, we expected lower values in reference tissue (higher values before transformation). Because feature values in repair and adjacent tissue approached the reference at 24 months, we assume that GLCM analysis of T_2_ maps can show a positive effect of MACT on surrounding tissue.

In recent years, several articles about GLCM analysis of knee cartilage have been published. Although these articles concern osteoarthritis (OA), there may be some similarities between OA lesions and MACT grafts that have not fully maturated, namely, in elevated T_2_.

Carballido-Gamio [22] reported elevated correlation, homogeneity, and entropy in osteoarthritic cartilage in the direction parallel to the cartilage surface between patient follow-ups. Joseph et al [19] reported that contrast was elevated in individuals with risk factors for OA. Schooler et al [21] performed a study with similar GLCM parameters (offset = 1, angle = 0° and 90°, flattened ROI) in patients with OA and healthy volunteers and they found elevated contrast 1, 2, and 3 years after baseline measurement and elevated entropy in the direction parallel to the cartilage. Heilmeier et al [41] reported an up to 40% higher risk for total knee replacement with a one-SD increase in contrast. Blumenkrantz et al reported elevated entropy in OA patients [24].

There are also few limitations that have to be considered. The number of cases was relatively low (*n* = 23). The first examination was done 12 months after MACT. At that time, the MACT transplant is expected to be fully matured and should not be as highly hydrated as it is less than six months after surgery. Therefore, a more rapid change in texture that could have occurred prior to the measurements in this study would have been missed. Due to the relatively large size of the repair tissue, reference cartilage was often chosen near the magic angle, but the overall increase of signal should not have affected the texture. The texture analysis was performed on three consecutive slices and not in a 3D manner, because of the non-zero slice distance used in multi-echo spin-echo T_2_ mapping.

## Conclusion

In conclusion, GLCM texture analysis is a useful add-on to T_2_ mapping in the evaluation of knee cartilage after MACT by increasing the sensitivity to changes in cartilage structure. Some texture features, namely, correlation, homogeneity, contrast, and difference entropy, changed significantly in repair tissue and adjacent tissue between 12 and 24 months after surgery, indicating a sizable change in texture, and likely, collagen structure. The results of this study, in accordance with previous research, suggest that the use of texture analysis provides additional information not only about the state and maturation of the repair tissue but also about the texture and composition of the repair cartilage and adjacent morphologically normal-appearing cartilage tissue.

## Supplementary Information

ESM 1 (DOCX 33 kb)

## Abbreviations

GLCM: Gray-level co-occurrence matrix
GRE: Gradient echo sequence
ICRS: International Cartilage Regeneration and Joint Preservation Society
MACT: Matrix-associated autologous chondrocyte transplantation
MFx: Bone marrow stimulating techniques
MOCART: Magnetic resonance observation of cartilage repair tissue
MRI: Magnetic resonance imaging
OA: Osteoarthritis
OCD: Osteochondritis dissecans
REML: Restricted maximum likelihood
ROI: Region of interest

## Declarations

### Guarantor

The scientific guarantor of this publication is MSc. Vladimir Juras, PhD.

### Methodology

• prospective

• observational

• multicenter study
