# Supplementary material for: Adjacent cartilage tissue structure after successful transplantation: a quantitative MRI study using T2 mapping and texture analysis
Source: Eur Radiol. 2022 Jun 23;32(12):8364–75. doi: 10.1007/s00330-022-08897-y (PMC9705472; doi:10.1007/s00330-022-08897-y)
Supplement: Supplementary file 1 — (DOCX 33 kb) [file 330_2022_8897_MOESM1_ESM.docx]

**Appendix A.** Analyzed GLCM features

| **Feature** | **Formula** | **Description** | **Reference** |
| --- | --- | --- | --- |
| *Autocorrelation* | $\sum_{i=1}^{N} \sum_{j=1}^{N} (i\cdot j)p(i,j)$ | Represents the extent of pattern repetition and periodically changes, depending on the repetitiveness of the pattern. | [1] |
| *Correlation* | $\sum_{i=1}^{N} \sum_{j=1}^{N} \frac{(i\cdot j)p(i,j)-\mu_{x}\mu_{y}}{\sigma_{x}\sigma_{y}}$ | Correlation is calculated differently from the other texture measures. As a result, it gives different information, and therefore, is more independent of other features. GLCM feature correlation shows correlation r^2^ < 0.5 with other features. Its values are always between -1 and +1 and it provides information about the predictable and linear relationship between the two neighboring pixels. | [2, 3] |
| *Homogeneity* | $\sum_{i=1}^{N} \sum_{j=1}^{N} \frac{p(i,j)}{1+{(i-j)}^{2}}$ | It is a measure of the homogeneity of an image, with more uniform gray levels, resulting overall in a higher overall value. | [1, 2] |
| *Contrast* | $\sum_{i=1}^{N} \sum_{j=1}^{N} {(i-j)}^{2}p(i,j)$ | Contrast is a measure of the local intensity variation. A larger value correlates with a greater disparity in intensity values among neighboring pixels. | [2] |
| *Difference entropy* | $-\sum_{k=0}^{N-1} p_{x-y}(k) log p_{x-y}(k)$ | Difference entropy belongs to measures related to orderliness and is similar to entropy. It is a measure of the randomness/variability in value differences between neighboring pixels. | [2, 3] |

Where $p\left( i,j \right),$ the normalized co-occurence matrix is equal to $\frac{P(i,j)}{\sum P(i,j)}$ with $P(i,j)$ being the $(i,j)$-th entry of the computed GLCM; N is the total number of gray levels in the image; and $\mu_{x}$, $\mu_{y}$ and $\sigma_{x}$, $\sigma_{y}$ denote the mean and standard deviations of the row and column sums of the GLCM, respectively.

The gray level difference distribution is defined as $p_{x-y}\left( k \right)= \sum_{i=1}^{N} \sum_{j=1}^{N} p\left( i,j \right), k =0, 1, 2\ldots, N-1$

**Appendix B.** Summary of linear mixed effects model of T_2_ values in repair, adjacent, and reference tissue at 12 and 24 months after surgery

|  | **Mean** T_2_ | | |
| --- | --- | --- | --- |
| *Predictors* | *Estimates* | *CI* | *p* |
| Intercept* (T_2_) | 56.59 | 53.96 – 59.22 | **<0.001** |
| Adjacent tissue | -3.34 | -7.53 – 0.85 | 0.118 |
| Reference tissue | -2.26 | -5.54 – 1.03 | 0.178 |
| Time** | -5.82 | -8.23 – -3.41 | **<0.001** |
| Adjacent tissue:Time** | 4.69 | 1.29 – 8.10 | **0.007** |
| Reference tissue:Time** | 4.98 | 1.58 – 8.39 | **0.004** |
| **Random Effects** | | | |
| σ^2^ | 17.37 | | |
| τ00 Case | 24.05 | | |
| τ11 Case - Adjacent tissue | 70.19 | | |
| τ11 Case - Reference tissue | 29.91 | | |
| ρ_01_ | -0.72 | | |
|  | -0.37 | | |
| ICC | 0.64 | | |
| N Case | 23 | | |
| Observations | 138 | | |
| Marginal R^2^ / Conditional R^2^ | 0.064 / 0.665 | | |
| * Reference tissue at 12 months; ** Time-point 24 months |  | | |

**Appendix C.** Summary of linear mixed effects models of selected GLCM features in repair, adjacent, and reference tissue at 12 and 24 months after surgery

| **Autocorrelation** | | | | **Homogeneity** | | | | | **Correlation ^3^** | | | | | **∛Contrast** | | | | | **Difference entropy** | | | | |
| --- | --- | --- | --- | --- | --- | --- | --- | --- | --- | --- | --- | --- | --- | --- | --- | --- | --- | --- | --- | --- | --- | --- | --- |
| *Predictors* | *Estimates* | *CI* | *p* | | *Predictors* | *Estimates* | *CI* | *p* | | *Predictors* | *Estimates* | *CI* | *p* | | *Predictors* | *Estimates* | *CI* | *p* | | *Predictors* | *Estimates* | *CI* | *p* |
| Intercept* (Autocorrelation) | 76.37 | 65.57 – 87.17 | **<0.001** | | Intercept* (Homogeneity) | 0.69 | 0.66 – 0.72 | **<0.001** | | Intercept* (Correlation^3^) | 0.55 | 0.47 – 0.63 | **<0.001** | | Intercept* (∛Contrast) | 0.99 | 0.93 – 1.05 | **<0.001** | | Intercept* (Difference) | 1.06 | 0.99 – 1.13 | **<0.001** |
| Time** | -5.14 | -10.84 – 0.56 | 0.077 | | Time** | -0.02 | -0.04 – -0.01 | **0.001** | | Time** | -0.06 | -0.09 – -0.03 | **<0.001** | | Time** | -0.08 | -0.11 – -0.05 | **<0.001** | | Time** | 0.08 | 0.05 – 0.12 | **<0.001** |
| Adjacent tissue | 32.82 | 22.34 – 43.30 | **<0.001** | | Adjacent tissue | -0.03 | -0.06 – -0.004 | **0.027** | | Adjacent tissue | -0.03 | -0.13 – 0.07 | 0.56 | | Adjacent tissue | -0.05 | -0.11 – 0.01 | 0.075 | | Adjacent tissue | 0.02 | -0.05 – 0.09 | 0.546 |
| Reference tissue | 49.11 | 35.40 – 62.83 | **<0.001** | | Reference tissue | -0.07 | -0.11 – -0.02 | **0.002** | | Reference tissue | -0.17 | -0.30 – -0.03 | **0.015** | | Reference tissue | -0.14 | -0.21 – -0.06 | **<0.001** | | Reference tissue | 0.11 | 0.001 – 0.21 | **0.047** |
| **Random Effects** | | | | | **Random Effects** | | | | | **Random Effects** | | | | | **Random Effects** | | | | | **Random Effects** | | | |
| σ^2^ | | 291.88 | | | σ^2^ | | 0.01 | | | σ^2^ | | 0 | | | σ^2^ | | 0.01 | | | σ^2^ | | 0.01 | |
| τ00 Case | | 503.62 | | | τ00 Case | | 0.004 | | | τ00 Case | | 0.03 | | | τ00 Case | | 0.01 | | | τ00 Case | | 0.03 | |
| τ11 Case - Adjacent tissue | | 365.5 | | | τ11 Case - Adjacent tissue | | 0.004 | | | τ11 Case - Adjacent tissue | | 0.05 | | | τ11 Case - Adjacent tissue | | 0.01 | | | τ11 Case - Adjacent tissue | | 0.02 | |
| τ11 Case - Reference tissue | | 834.09 | | | τ11 Case - Reference tissue | | 0.01 | | | τ11 Case - Reference tissue | | 0.1 | | | τ11 Case - Reference tissue | | 0.02 | | | τ11 Case - Reference tissue | | 0.06 | |
| ρ_01_ | | -0.45 | | | ρ_01_ | | -0.29 | | | ρ_01_ | | -0.48 | | | ρ_01_ | | 0.35 | | | ρ_01_ | | -0.37 | |
|  | | -0.74 | | |  | | -0.59 | | |  | | -0.51 | | |  | | -0.08 | | |  | | -0.58 | |
| ICC | | 0.61 | | | ICC | | 0.74 | | | ICC | | 0.84 | | | ICC | | 0.76 | | | ICC | | 0.75 | |
| N Case | | 23 | | | N Case | | 23 | | | N Case | | 22 | | | N Case | | 19 | | | N Case | | 23 | |
| Observations | | 138 | | | Observations | | 138 | | | Observations | | 132 | | | Observations | | 114 | | | Observations | | 138 | |
| Marginal R^2^ / Conditional R^2^ | | 0.364 / 0.751 | | | Marginal R^2^ / Conditional R^2^ | | 0.117 / 0.774 | | | Marginal R^2^ / Conditional R^2^ | | 0.103 / 0.861 | | | Marginal R^2^ / Conditional R^2^ | | 0.130 / 0.794 | | | Marginal R^2^ / Conditional R^2^ | | 0.090 / 0.771 | |
| * Reference tissue at 12 months; ** Time-point 24 months | | | | | | |  | | |  | |  | | |  | |  | | |  | |  | |
|  |  |  |  |  |  |  |  | | |  | |  | | |  | |  | | |  | |  | |

**Appendix D.** Estimated values of T_2_ and selected GLCM features in repair, adjacent, and reference cartilage at 12 and 24 months based on mixed effects models.

| *Variable* | *Repair tissue at 12 months* | *95% CI* | *Repair tissue at 24 months* | *95% CI* | *Adjacent tissue at 12 months* | *95% CI* | *Adjacent tissue at 24 months* | *95% CI* | *Reference tissue at 12 months* | *95% CI* | *Reference tissue at 24 months* | *95% CI* |
| --- | --- | --- | --- | --- | --- | --- | --- | --- | --- | --- | --- | --- |
| **T_2_ [ms]** | 56.59 | 53.96 – 59.22 | 50.76 | 48.13 - 53.39 | 53.24 | 50.29 - 56.21 | 52.12 | 49.16 - 55.08 | 54.33 | 51.40 - 57.27 | 53.49 | 50.56 - 56.43 |
| **Autocorrelation** | 76.37 | 65.57 - 87.17 | 71.23 | 60.43 - 82.03 | 109.19 | 98.54 - 119.83 | 104.05 | 93.40 - 114.69 | 125.48 | 115.74 - 135.23 | 120.34 | 110.60 - 130.09 |
| **Homogeneity** | 0.69 | 0.66- 0.72 | 0.67 | 0.64 -0.70 | 0.66 | 0.63 - 0.69 | 0.64 | 0.60 - 0.67 | 0.63 | 0.59 - 0.66 | 0.60 | 0.57 - 0.64 |
| **Correlation^3^** | 0.55 | 0.47 - 0.63 | 0.49 | 0.41 - 0.57 | 0.52 | 0.43 - 0.61 | 0.46 | 0.37 - 0.55 | 0.38 | 0.26 - 0.50 | 0.32 | 0.20 - 0.44 |
| **∛Contrast** | 0.99 | 0.93 - 1.05 | 0.91 | 0.85 - 0.97 | 0.94 | 0.85 - 1.02 | 0.86 | 0.77 - 0.94 | 0.85 | 0.77 - 0.94 | 0.78 | 0.670- 0.86 |
| **Difference Entropy** | 1.06 | 0.99 - 1.13 | 1.14 | 1.07 - 1.22 | 1.08 | 1.00 - 1.15 | 1.16 | 1.09 - 1.24 | 1.17 | 1.08 - 1.25 | 1.25 | 1.16 - 1.33 |

**References**

1. Soh LK, Tsatsoulis C (1999) Texture analysis of sar sea ice imagery using gray level co-occurrence matrices. IEEE Trans Geosci Remote Sens 37:780–795. https://doi.org/10.1109/36.752194

2. Haralick RM, Dinstein I, Shanmugam K (1973) Textural Features for Image Classification. IEEE Trans Syst Man Cybern SMC-3:610–621. https://doi.org/10.1109/TSMC.1973.4309314

3. Hall-Beyer M (2017) GLCM Texture: A Tutorial v. 3.0 March 2017. https://prism.ucalgary.ca/handle/1880/51900. Accessed 23 Aug 2021
